# Some data quality issues at ClinicalTrials.gov

**DOI:** 10.1186/s13063-019-3408-2

**Published:** 2019-06-24

**Authors:** Neha Chaturvedi, Bagish Mehrotra, Sangeeta Kumari, Saurabh Gupta, H. S. Subramanya, Gayatri Saberwal

**Affiliations:** 1 Institute of Bioinformatics and Applied Biotechnology, Biotech Park, Electronics City Phase 1, Bengaluru, Karnataka 560100 India; 2 Present address: JP Morgan & Chase, Bengaluru, Karnataka India; 3 Present address: Institute of Biochemistry and Biology, University of Potsdam, Potsdam-Golm, Germany

**Keywords:** ClinicalTrials.gov, Drugs, Biologicals, Clinical trial, Principal Investigator, Data quality, Database errors

## Abstract

**Background:**

Clinical trial registries have been established as a form of public accountability. Sponsors ought to register their trials promptly and accurately, but this is not always done. Some of the problems include non-registration of trials, registration of trials with incomplete information, and non-reporting of trial results on time. In this study we enumerate or quantify some quality issues with respect to Principal Investigator (PI) and Responsible Party data.

**Methods:**

We analyzed interventional trials registered with ClinicalTrials.gov. Using certain selection criteria, we started with 112,013 records, and then applied further filters. The trial had to (a) start between 1 January 2005 and 31 December 2014, (b) include a “drug” or “biological” in the “intervention” field, (c) be registered with an American authority, and (d) list a real person’s name as investigator and also his or her role in the study.

**Results:**

We identified four categories of errors in the ClinicalTrials.gov records. First, some data were missing. The name of the investigator, or his or her role, was missing in 12% of 35,121 trials. In examining 71,359 pairs of names and roles, 17% of the “names” were found to be not those of real persons, but instead junk information. Second, there were variations in a large number of names. We identified 19 categories of variants. We determined that 13% of the names had variants that could not be resolved using a program. Third, some trials listed many PIs each, although only one such person holds overall responsibility for the trial and therefore not more than one person should be listed as PI. Fourth, in examining whether the PI’s name was available as part of the Responsible Party tag, we found that in 1221 (3.5%) of 35,121 trials, the Responsible Party tag is absent.

**Conclusions:**

We have outlined four categories of problems with data hosted by ClinicalTrials.gov and have quantified three of them. We also suggest how these errors could be prevented in future. It is important to carry out various kinds of audits of trial registries, in order to identify lacunae in the records, that they be addressed.

**Electronic supplementary material:**

The online version of this article (10.1186/s13063-019-3408-2) contains supplementary material, which is available to authorized users.

## Background

Clinical trials involve experiments on human beings and the results of many of them have implications for patient care. As such the reporting of trials must be prompt, accurate, and comprehensive. Sadly this is not always so and for some decades now there has been a push to raise the standard of reporting. Recent years have seen distinct improvements and these positive changes have come about due to various types of efforts. Since 1986 in particular there has been a call to establish registries, where sponsors register details of their trials [1]. Over the past two decades many registries have been established, including the Australian New Zealand Clinical Trials Registry (ANZCTR), Clinical Trial Registry-India (CTRI), the European Union Clinical Trial Registry, the German Clinical Trials Register, the Japan Primary Registries Network, and the United States’ ClinicalTrials.gov. The International Committee of Medical Journal Editors (ICMJE) has also pressured researchers to register their trials. Since 13 September 2005, it has required that trials intended for publication in their journals need be registered in a publicly accessible database before the recruitment of patients begins [2]. Subsequent to the announcement of this policy, there was a substantial increase in the number of trials registered with ClinicalTrials.gov [3].

Although trials have often been registered retrospectively, several steps have been taken to push for prospective registration [4–7]. This would ensure that it is possible to verify, for instance, that (a) the results of all trials have been reported and that trials with positive outcomes have not been selectively reported; and (b) there is no difference in the protocol, in the number or definitions of the primary and secondary outcomes, or in the strategy for data analysis between the information in the registry and that in the subsequent academic publication [8].

As a form of public accountability it is of paramount importance that the results of individual trials be made public. This also enables scrutiny of trial data by people unconnected with the trial in any way, and therefore without bias. However, it is important to note that ClinicalTrials.gov and other registries have been used to perform a variety of other analyses. These include (a) determining what the size of a trial tends to be and what kinds of trial methodologies are adopted by industry and non-industry sponsors [9]; (b) for globalized trials, determining the distribution of trial leadership over different nations [10]; (c) analyzing why trials have been terminated prematurely [11]; (d) enumerating which organizations sponsor trials, how many trials each sponsor conducts, and where these trial sponsors are located globally [12]; (e) profiling the clinical trial landscape of a country [4]; and (f) examining how the funders of clinical trials change over time [13]. Such analyses have policy implications since they may, for instance, help a government understand (a) what kinds of trials are ongoing in the country and whether they are relevant to the nation’s health burden; (b) whether a large fraction of trials are sponsored by local institutions or whether the country is simply a low-cost destination for trials sponsored by foreign organizations; and (c) how many trials a given Principal Investigator (PI) has conducted and whether there is cause for concern on this score. As such, trial registries serve a greater purpose than merely holding data pertaining to individual trials.

Registries have transformed the landscape of trial reporting. In India, for instance, the reporting of methods was better on CTRI than in academic publications [14]. Nevertheless, all is not well with the registries and some of the problems are as follows: (a) all trials that are required by law to be registered may not be [15]; (b) trials may be registered with incomplete information [16, 17]; (c) for a multi-country trial registered with different registries there may be discrepancies in the data in these registries [18]; (d) the results of a trial may not be reported within a year of completion, as is required in the USA, for instance [19]; and (e) a registry may be misused to market an unapproved procedure as a trial [20].

The present study arose from a research question inspired by a ruling regarding trials in India. In 2012, Indian Principal Investigators (PIs) were barred from running more than three trials at a time [21]. Any discussion of the optimal number should be based on the norms in countries with the best regulations. In order to understand whether this restriction—since revoked—was justified, we wished to look at the situation in the US where the largest number of trials is conducted [22]. During the work we realized that it was not possible to answer this question, partly due to issues with the quality of the data in ClinicalTrials.gov. We have therefore quantified or enumerated some of these data quality issues which pertain solely to the PI or Responsible Party (RP; which may list the PI). As such, this study is in the nature of an audit of these two fields.

## Methods

We used data hosted by ClinicalTrials.gov, the largest registry of clinical trials in the world [23]. We accessed http://clinicaltrials.gov on 14 October 2016 and did an advanced search, with certain filters, as follows. For “Study type” we chose “Interventional studies”. For “Recruitment status” we considered the following categories: (a) Active, not recruiting, (b) Completed, (c) Enrolling by invitation, (d) Recruiting, (e) Suspended, and (f) Terminated. We then selected “Phase” 0–4 and “Record first received” 1/1/2005 to 12/31/2014. This yielded a total of 112,013 records (each with a unique NCT ID), which were downloaded in six lots, corresponding to the categories (a)–(f) above. The data were processed in these six lots for several steps before being merged into a single file. To be noted is that the 112,013 XML files and Additional file 3: Table S1, Additional file 4: Table S2, Additional file 5: Table S3, and Additional file 6: Table S4 are large files and have therefore been hosted at https://osf.io/jcb92. The scripts used for particular steps are provided in Additional file 2: S1 Folder, also available at https://osf.io/jcb92. A summary of the first set of steps taken to process the data is provided in Table 1 and additional details of the methodology are in Additional file 1: S1 Text.

**Table 1:** Steps taken to process the data

1a. Steps taken to process the data with each record representing one trial				
Number	Processing step	Records selected	Records rejected	Table(s) with the details
1	Downloaded files	112,013		Additional file 3: Table S1
2	Selected trials with the start date between 1/1/2005 and 12/31/2014	79,838	32,175	Additional file 4: Table S2
3	Selected trials with “drug:” or “biological:” in the intervention field	64,496	15,342	Additional file 5: Table S3
4	Selected trials with a completion date or primary completion date	63,786	710	Additional file 6: Table S4 and Additional file 7: Table S5
5	Selected trials registered with a US authority	35,121	28,665	Additional file 8: Table S6 and Additional file 9: Table S7
6	Selected trials that listed both the investigator’s name and role	31,392	3729	Additional file 10: Table S8
7	Selected trials where the number of investigators’ names matched the number of their roles	31,375	17	Additional file 11: Table S9
1b. Steps taken to process the data, with each row in Additional file 12: Table S10 and Additional file 13: Table S11 representing one investigator				
Number	Processing step	Names selected	Names rejected	Table(s) with the details
8	In trials where there were multiple names, separated them to create pairs of single names and their corresponding roles	71,359		Additional file 12: Table S10
9	Selected trials where the investigator’s name was that of a real individual	60,787	10,572*	Additional file 13: Table S11

*These 10,572 “names” came from 8907 NCT IDs

From the 112,013 records (Additional file 3: Table S1) we first selected those that had a start date from 1/1/2005 to 12/31/2014 (both inclusive). This yielded 79,838 records (Additional file 4: Table S2). From these, we selected those that contained “drug:”, “biological:”, or both of these terms in the intervention field. This yielded 64,496 “medicine” trials (Additional file 5: Table S3). We then examined the studies for their completion dates. This date had to be listed, but did not have to be in the 10-year window. If there was “null” for the “completion date”, but a valid entry for the “primary completion date”, the record was selected. We rejected the record if it lacked both these dates. This step yielded 63,786 records (Additional file 6: Table S4 and Additional file 7: Table S5), which were bifurcated into those that were registered with at least one authority in the US (a total of 35,121 records in the Additional file 8: Table S6 and Additional file 9: Table S7) and those that were not. Examples of such authorities are listed in Additional file 1: S1 Text.

We then processed the 35,121 records to identify those that listed both the name of the investigator and his or her role in the trial. This yielded 31,392 records (Additional file 10: Table S8) that contained both the names and the corresponding roles, and 3729 records that were missing one or both pieces of information. Many of the 31,392 records had multiple names, and in 17 cases the number of names did not match the number of roles. We rejected these 17 (Additional file 11: Table S9) and took forward the remaining 31,375 records.

The next step was to process records that contained multiple names and roles such that there were two columns, with each row containing one name and the corresponding role. This yielded 71,359 pairs of names and roles (Additional file 12: Table S10). In many of them the names of real persons were substituted with “non-person” junk information such as designations, call center numbers, and so on. We rejected 10,572 rows and took forward the 60,787 rows (Additional file 13: Table S11) that had the names of real persons. To be noted is that the 10,572 names have 8907 unique NCT IDs (Additional file 14: Table S12). We wished to determine the frequency of occurrence of a person’s name in these 60,787 rows but, on examining the names, identified many problems that prevented this.

## Results

As mentioned above, we used various criteria to create a well-defined set of 35,121 trials, which we processed to yield 71,359 pairs of investigators’ names and their roles. We wished to determine the frequency of occurrence of individual names in these records but discovered that many “names” were junk information which prevented any meaningful assessment of the number of PIs or their frequency. Overall, we encountered four categories of errors with PI (or RP) information in ClinicalTrials.gov data as detailed below.

### Missing data

In two of the several steps of data processing we found that a notable amount of data was missing. First, in trying to match name and role we found that one or both pieces of information were missing in 3729 (11.9%) of the 35,121 trial records (Table 1). Also, in 17 cases the number of names and number of roles in a given trial record did not match (Table 1). Second, since a given record may have more than one name and role, subsequent processing led us to a list of 71,359 pairs of names and roles. In 10,572 (17.4%) of these (Table 1), the “name” field contained junk information instead of the name of a real person. Examples of this “non-person” junk information were Bioscience Center, Central Contact, Chief Development Officer, Chief Medical Officer, Clinical Development Manager, Clinical Development Support, Clinical Director Vaccines, Clinical Program Leader, Clinical Project Lead, Clinical R&D, Clinical Sciences & Operations, Clinical Study Operations, Clinical Trial Management, Clinical Trials, [company’s call center number], [company’s name], Global Clinical Registry, Investigational Site, MD, Medical Director, Medical Director Clinical Science, Medical Monitor, Medical Responsible, Professor, Program Director, Sponsor’s Medical Expert, Study Physician, TBD TBD, Use Central Contact, Vice President Medical Affairs, VP Biological Sciences, and VP Clinical Science Strategy. After removing such junk “names”, we were left with 60,787 pairs of names and roles.

For the rejected records of both the Additional file 10: Table S8 and the Additional file 13: Table S11, we also wished to determine whether the PI had, at any point, been listed during the history of the trial. To do this we examined the history of a sample of records (Additional file 1: S1 Text and Additional file 15: Table S13). We used a 5% sample each of NCT IDs of the 3729 rejects of the Additional file 10: Table S8 and of the 8907 unique NCT IDs rejected in the Additional file 13: Table S11 (Additional file 14: Table S12), which amounted to 211 and 422 trials, respectively. We found that only 16 (7.5%) out of 211 Additional file 10: Table S8 rejects and only 9 (2%) out of 422 Additional file 13: Table S11 rejects had a PI in at least one history record. Overall, this amounted to 25 of 633, or 4% of the rejects overall. Taking into account these percentages, 3729 rejects of the Additional file 10: Table S8 are reduced to 3449, and 8907 rejects of the Additional file 13: Table S11 are reduced to 8729.

Finally, we summarized the data above. The overall number of records with missing or junk information was as follows: (a) 3449/35,121 in Additional file 10: Table S8; (b) 17/35,121 in Additional file 11: Table S9; and (c) 8729/35,121 in Additional file 13: Table S11. These add up to 12,195/35,121 (35%) of NCT IDs with missing or junk information in the PI field.

### Variations in names

Next we wished to determine the frequency with which a given person’s name appeared as the PI in the set of 60,787 names in Additional file 13: Table S11. It turned out that, of the 60,787 names, 82% were those of a PI, with the rest being those of sub-investigators (5%), Study Directors (9%), and Study Chairs (4%). For the purpose of the results described below, however, this variety of designations did not matter. We took several steps to clean up the names to ensure that each individual was represented by a single name. However, there were different categories of problems with respect to the way names were entered in the database which made this process challenging. These issues are listed in 18 categories below.

- Extraneous information along with the name:
  (i) The name may have had a prefix (e.g., Prf., Prof. Dr., COL) or suffix (e.g., MD; Jr.; III; M.D., Principal Investigator; BSc, MBCHB, MD, Study Director) of varying lengths.
  (ii) The name may have included a punctuation mark.
- Different kinds of variations of the name:
  (i) The name may have had spelling mistakes.
  (ii) One or more parts of the name may have been abbreviated or truncated.
  (iii) Parts of the name may have been ordered differently.
  (iv) The middle name may or may not have been mentioned.
  (v) Parts of the name may or may not have been hyphenated.
  (vi) The surname may have been modified.
  (vii) The surname may have been repeated.
  (viii) The person’s initials may or may not have been separated by spaces.
  (ix) The entire name, or parts of it, may have been in capitals.
  (x) The name may have contained a non-English character or the closest English character.
  (xi) The first name may have been split into two, or the first and middle name may have been merged.
  (xii) The surname may have been split into two, or the middle and surname may have been merged.
  (xiii) A nickname, in brackets, may have been mentioned in the middle of the name.
  (xiv) The Americanized nickname of part of a foreign name may have replaced the original.
- Other complications with the names:
  (i) A person’s entire name may have been represented by just one word.
  (ii) Two individuals may have shared the same name.

We went on to eliminate or quantify categories a(i, ii), b (iv, ix, xiii) and c(i) (Additional file 1: S1 Text and Additional file 16: Table S14, Additional file 17: Table S15, Additional file 18: Table S16, Additional file 19: Table S17 and Additional file 20: Table S18), and obtained an estimate of 12.8% of names that could not be identified unambiguously. Although we have not quantified the other categories of errors, based on preliminary work, we believe that they are not numerous.

### Multiple PIs per trial

Another category of error concerned trials that listed more than one person as PI. Examples included NCT01954056 (with 18 PIs), NCT00405704 (21 PIs), NCT01819272 (50 PIs), NCT00419263 (73 PIs), and NCT01361308 (74 PIs).

### Missing RP tag

Finally we wished to know whether PI information was available from the RP tag. For this, we examined the 35,121 records from Additional file 10: Table S8. We found that the RP tag was missing in 1221 (3.5%) of 35,121 records (Additional file 21: Table S19). As explained in Additional file 1: S1 Text, the RP details were usually provided both at the top of the NCT ID record and at the bottom. At the top, the exact wording was usually “Information provided by (Responsible Party):...”. However, in 1221 records the wording was “Information provided by:...”. These records did not have the RP information at the bottom either. Thus, anybody using automated methods to search for RP information based on the RP tag would not find it.

In terms of whether the RP field was useful to obtain PI information, we used a sample of 500 records and found PI information only in 138 of them (Additional file 21: Table S19). All of these cases already had PI information, as determined in Additional file 10: Table S8. Thus, the RP field did not yield any additional PI information.

## Discussion

As discussed above, data in clinical trial registries is often repurposed. It may be useful to know the number of unique PIs, and their frequency of occurrence, in trials registered with ClinicalTrials.gov. If, as we set out to do, one wished to identify all the PIs and then count their frequency, one had to go to great lengths to process the data. The many challenges we encountered are enumerated above.

The first challenge concerned missing PI data. Following from the Federal regulations outlined in 42 CRF Part 11, and as detailed at https://prsinfo.clinicaltrials.gov/definitions.html, it is not mandatory to list each of the scientific leadership, such as the PI, by name and role while registering a trial with ClinicalTrials.gov. The sponsor is the Responsible Party, and it is mandatory to list the sponsor, the sponsor-investigator, or the PI designated by the sponsor as the RP. Despite this, the PI or other scientific leadership’s name and role fields are filled for most trials. In some cases, however, junk information is provided instead. Given that trial data are repurposed for other kinds of analyses, it would make trial records even more valuable if these data were captured.

The non-registering and non-reporting of trials has become a high profile issue, with the naming and shaming of sponsors that do not register their trials or do not report trial results on time [19, 24, 25]. However, even for trials that are registered there are quality issues that we need to be concerned about. Examples of errors previously noted in such registries include (a) observational or other kinds of trials that were labeled as interventional trials [26] and (b) the non-listing of trial sites both at the start of a study and even after its completion [16]. In contrast, a recent analysis of over 10,000 Australian trials registered either with ANZCTR or ClinicalTrials.gov noted that data regarding the primary sponsor was missing for just one trial each on the two registries [4]. This is an example of how low the error rate could be for a given field of information, although it ought to be possible to ensure complete accuracy. Were the filing of PI information to be made mandatory, the current errors could be brought down to nil.

Aside from missing data, there were problems with regard to names due to both junk information and variations in a given PI’s name. With 35% of the NCT IDs lacking PI information or containing junk information in this field, and about 13% of PI names that are ambiguous, overall about half of the NCT IDs do not contain PI names that could readily be repurposed.

For the sake of accuracy it is important that there be just one version of a person’s name which should unambiguously identify that person. The registration system should be modified so that a person’s name has to be separately registered, and thereafter only registered names can be chosen in the “name” field. A person’s name may change with time, and so it should be possible to choose a registered name and also list the current name. This should be supplemented with a unique and permanent ID such as the Open Researcher and Contributor ID (ORCID). Only valid ORCID numbers should be accepted by the system and the database should not permit the registration of a trial unless the name of a PI—in these standardized formats—has been entered. Other researchers have also noted the absence of proper information in the name field [26, 27] and clearly the situation has not improved much over time. If, as stated several years ago [26], it is important that the scientific leadership of a trial be named, then those names must be accurate. Further, each name needs to be in a standardized format to permit an automated method of determining the frequency of its occurrence in a given registry or across registries. This is the only way to ensure full transparency of this part of the database.

We now come to the issue of a trial listing multiple PIs. Since a PI is defined as “The person who is responsible for the scientific and technical direction of the entire clinical study” [28], each trial should list just one overall PI. Trials may have multiple sites, each with its own PI, and therefore there may be a large number of site PIs. The trial summary may therefore list several PIs under “Study Locations”, but must not list more than one person in the (overall) “Investigator” field. As such, during the registration process, ClinicalTrials.gov should not permit the entry of more than one name as overall PI for the trial.

Although, in principle, PI information may be available from the RP field, we note that there are complications in identifying the RP easily. These are as follows: (a) there is an RP field both at the top and bottom of an NCT record, and information may be missing in either of them; (b) as discussed above, and surprisingly, the RP tag may be missing altogether at the top of the NCT record; (c) retrieving information from the RP at the bottom of the NCT record is non-trivial due to the non-uniform manner in which the information is stored; (d) the details of the RP are not necessarily identical in these two fields (although they do refer to the same organization). Future versions of ClinicalTrials.gov should aim to solve these issues.

Aside from correcting or preventing obvious errors in ClinicalTrials.gov, a bigger issue is the need to validate all the information in the database. As Dr. Scott Gottlieb, the former Commissioner of the US Food and Drug Administration (FDA), admitted, it is challenging to link trial information registered with ClinicalTrials.gov and the relevant application to the FDA for approval of a candidate drug [29]. It would aid transparency if it was mandatory to list the NCT IDs of the relevant clinical trials in these applications to the FDA.

Another issue concerns trials registered in multiple registries. In such cases, certain information should match in all the records related to a given trial [18]. However, there is no easy way to verify that it is really the same. In the interest of complete truthfulness and transparency, it is important that data in all registries are regularly and thoroughly cross-validated.

Given the fact that there are many trial registries, and each has many fields of information that need to be correctly filled, the limitation of our study is that it was restricted to one registry and only examined the PI and RP fields.

## Conclusions

Registries were created in order to fulfill the ethical and scientific objectives of reporting on all trials promptly, completely, and accurately. However, the very objectives of creating a registry are defeated if trials are not registered or the registered data have substantial errors. As such there is increasing emphasis both on the importance of registering trials [30] and on improving the quality of data in the registries [31]. For trials registered with ClinicalTrials.gov, we have outlined four categories of problems with the names or roles of the PIs, or with the RP information, and have quantified three of them. We have also suggested how these errors could be prevented in future. Other researchers may wish to conduct additional audits of the database to identify or quantify other categories of errors in the hosted data.

## Additional files

Additional file 1: **S1.** Text: Further details of methodology. (DOC 62 kb)

Additional file 2: **S1.** Folder: The scripts used to process particular steps. The folder is available at https://osf.io/jcb92. (ZIP 10 kb)

Additional file 3: Data (112,013 records) comprises a spliced version of the 26 fields in tsv format and one field obtained from the XML files. The data are presented in the following six Recruitment Type categories: (1) Active, not recruiting (11,094 records), (2) Completed (67,294), (3) Enrolling by invitation (1022), (4) Recruiting (23,223), (5) Suspended (597), and (6) Terminated (8783). The sheets are numbered 1–6, respectively. The file is available at https://osf.io/jcb92. (ZIP 4860 kb)

Additional file 4: Records selected for their start dates. Records having a “Start date” from 1 January 2005 to 31 Deccember 2014 (both inclusive) are listed in a “StartDate” sheet, with the remaining records in a “StartDate_leftovers” sheet. The data (112,013 records from Additional file 3: **Table S1**) are presented in the following six Recruitment Type categories: (1) Active, not recruiting (8582 selected records, with 2512 leftovers), (2) Completed (50,012; 17,282), (3) Enrolling by invitation (606; 416), (4) Recruiting (12,991; 10,232), (5) Suspended (432; 165), and (6) Terminated (7215, 1568). The sheets are numbered 1–6, respectively. The file is available at https://osf.io/jcb92. (ODS 3850 kb)

Additional file 5: Records of drugs or biologicals. Records with “drug:”, “biological: ”, or both of these words in the “intervention” field are listed in a “Drugs+Bio” sheet, with the remaining in a “Drugs+Bio_leftovers” sheet. The data (79,838 records from Additional file 4: **Table S2**) are presented in the following six Recruitment Type categories: (1) Active, not recruiting (6742 selected records with 1840 leftovers), (2) Completed (41,451; 8561), (3) Enrolling by invitation (386; 220), (4) Recruiting (9379; 3612), (5) Suspended (339; 93), and (6) Terminated (6199; 1016). The sheets for these categories are numbered 1–6, respectively. The file is available at https://osf.io/jcb92. (ODS 2830 kb)

Additional file 6: Records with a completion date. The data (64,496 records from Additional file 5: Table S3) were sorted into a “ComplDate” sheet for trials with completion dates, with the remaining records in a “ComplDate_leftovers” sheet. The data are presented in the following six Recruitment Type categories: (1) Active, not recruiting (5471 selected records with 1271 leftovers), (2) Completed (38,997; 2454), (3) Enrolling by invitation (341; 45), (4) Recruiting (7833; 1546), (5) Suspended (233; 106), and (6) Terminated (5810; 389). The sheets for these categories are numbered 1–6, respectively. The file is available at https://osf.io/jcb92. (ODS 2300 kb)

Additional file 7: Records with a primary completion date. The data (58,685 records from Additional file 6: Table S4) were sorted into a “PriComplDate” sheet for trials with primary completion dates, with the remaining records in a “PriComplDate_leftovers” sheet. The data are presented in the following six Recruitment Type categories: (1) Active, not recruiting (1220 selected records with 51 leftovers), (2) Completed (2040; 414), (3) Enrolling by invitation (42; 3), (4) Recruiting (1382; 164), (5) Suspended (91; 15), and (6) Terminated (326; 63). The sheets for these categories are numbered 1–6, respectively. (XLS 7227 kb)

Additional file 8: Records with a completion date and registered with an authority in the USA. The data (58,685 records from Additional file 6: Table S4) were sorted into a “USA_ComplDate” sheet for trials registered with at least one authority in the US, and a “USA_ComplDate_leftovers” sheet with the remaining records. The data are presented in the following six Recruitment Type categories: (1) Active, not recruiting (3350 selected records with 2121 leftovers), (2) Completed (21,030; 17,967), (3) Enrolling by invitation (166; 175), (4) Recruiting (3167; 4666), (5) Suspended (134; 99), and (6) Terminated (3986; 1824). The sheets for these categories are numbered 1–6, respectively. (XLS 6129 kb)

Additional file 9: Records with a primary completion date and registered with an authority in the USA. The data (5101 records from Additional file 7: Table S5) were sorted into a “USA_PriComplDate” sheet for trials registered with at least one authority in the US, and a “USA_PriComplDate_leftovers” sheet with the remaining records. The data are presented in the following six Recruitment Type categories: (1) Active, not recruiting (1085 selected records with 135 leftovers), (2) Completed (1100; 940), (3) Enrolling by invitation (19; 23), (4) Recruiting (773; 609), (5) Suspended (59; 32), and (6) Terminated (252; 74). The sheets for these categories are numbered 1–6, respectively. (XLS 493 kb)

Additional file 10: Records that list both the name and the role of the investigator. The names of the investigators and their corresponding roles were extracted from Additional file 8: Table S6 (31,833 records) and Additional file 9: Table S7 (3288 records). They were sorted into a “NameAndRole” sheet for trials that listed both the name and role of the investigator, and a “Nulls” sheet that listed the remaining records. The data are presented in the following six Recruitment Type categories: (1) Active, not recruiting (4052 selected records with 383 leftovers), (2) Completed (19,404; 2726), (3) Enrolling by invitation (162; 23), (4) Recruiting (3784; 156), (5) Suspended (182; 11), and (6) Terminated (3808; 430). The sheets for these categories are numbered 1–6, respectively. (XLS 6779 kb)

Additional file 11: Records that list matching numbers of names and roles of the investigators. The data (31,392 records from Additional file 10: Table S8) were sorted into the “MatchedPipes” sheet, where the number of pipes (each one of which delineates one name or role) was the same in the name and corresponding role cells, and an “UnmatchedPipes” sheet with the remaining records. The data are presented in the following six Recruitment Type categories: (1) Active, not recruiting (4051 selected records with 1 leftover), (2) Completed (19,392; 12), (3) Enrolling by invitation (162; 0), (4) Recruiting (3782; 2), (5) Suspended (181; 1), and (6) Terminated (3807; 1). The sheets for these categories are numbered 1–6, respectively. (XLS 6429 kb)

Additional file 12: Data in the form of individual investigators and their matching roles. The data (31,375 records from Additional file 11: Table S9) were sorted into a “LineByLine” sheet, where records that contained multiple names and roles were segregated such that each row contains one name and the corresponding role. The data are presented in the following six Recruitment Type categories: (1) Active, not recruiting (4805 records), (2) Completed (24,135), (3) Enrolling by invitation (201), (4) Recruiting (37,248), (5) Suspended (214), and (6) Terminated (4756). The sheets for these categories are numbered 1–6, respectively. (XLS 5686 kb)

Additional file 13: Data with real persons. The data (71,359 records from Additional file 12: Table S10) were sorted into a “Person” sheet with the records that had the names of real people in the “Last name” field, and a “NonPerson” sheet with the remaining junk records. The data are presented in the following six Recruitment Type categories: (1) Active, not recruiting (4112 selected records with 693 leftovers), (2) Completed (17,081; 7054), (3) Enrolling by invitation (190; 11), (4) Recruiting (35,447; 1801), (5) Suspended (206; 8), and (6) Terminated (3751; 1005). The sheets for these categories are numbered 1–6, respectively. (XLS 5690 kb)

Additional file 14: The 10,572 rejected rows in Additional file 13: Table S11 came from 8907 unique NCT IDs. (ODS 203 kb)

Additional file 15: PIs in the history records of a sample of Additional file 10: Table S8 rejects and Additional file 13: Table S11 rejects. (ODS 219 kb)

Additional file 16: Processing of PI names to identify ambiguities. An overview of Additional file 17: Table S15 to Additional file 20: Table S18. (ODS 22 kb)

Additional file 17: Processing of PI names to identify ambiguities—step 1. (XLSX 3330 kb)

Additional file 18: Processing of PI names to identify ambiguities—step 2. (XLSX 1406 kb)

Additional file 19: Processing of PI names to identify ambiguities—step 3. (XLSX 2105 kb)

Additional file 20: Processing of PI names to identify ambiguities—step 4. (XLSX 553 kb)

Additional file 21: Whether PI information is available from Responsible Party. (XLSX 1495 kb)

## Data Availability

The data downloaded from ClinicalTrials.gov and the datasets generated by the first few steps of processing these data are available at https://osf.io/jcb92. All other data generated or analyzed during this study are included in this published article and its Additional files.

## Abbreviations

ANZCTR: Australian New Zealand Clinical Trials Registry
ClinicalTrials.gov: ClinicalTrials.gov
CTRI: Clinical Trial Registry-India
FDA: US Food and Drug Administration
ID: Identifier
ORCID: Open Researcher and Contributor ID
PI: Principal Investigator
RP: Responsible Party
US: United States of America
