# Supplementary material for: Some data quality issues at ClinicalTrials.gov
Source: Trials. 2019 Jun 24;20:378. doi: 10.1186/s13063-019-3408-2 (PMC6591874; doi:10.1186/s13063-019-3408-2)
Supplement: Supplementary file 1 — S1. Text: Further details of methodology. (DOC 62 kb) [file 13063_2019_3408_MOESM1_ESM.doc]

**S1 Text. Further details of Methodology.**

We provide further details on different stages of the methodology as follows:

(A) Downloading the records from CT.gov. (Relates to S1 Table)

(B) Two cases in the sorting of US- versus non-US authority records. (Relates to S6–S7 Tables)

(C) Examples of authorities in the US where trials were registered. (Relates to S6–S7 Tables)

(D) Processing the trials for the step of matching 'Last name' and 'Role'. (Relates to S6–S8 Tables)

(E) Separating single names and their corresponding roles. (Relates to S9–S10 Tables)

(F) Descriptions of the 10 scripts used to process data (Relates to S1, S8 and S10 Tables)

(G) Issues with regard to multiple PIs per trial.

(H) Identifying PIs in the history records of a sample of S8 Table rejects and S11 Table rejects. (Relates to S13 Table)

(I) Quantifying name ambiguities (Relates to S14 Table-S18 Table)

(J) Whether PI information is available from Responsible Party (Relates to S19 Table)

**(A) Downloading the records from CT.gov**

This note relates to S1 Table.

We accessed CT.gov ([http://clinicaltrials.gov](http://clinicaltrials.gov/)) on 14 October 2016 and did an Advanced Search, using the following filters:

1. 'Study type': Interventional studies
2. Exclude unknown status
3. 'Phase': 0–4
4. 'Record first received': 1/1/2005 to 12/31/2014

[Note: The CT.gov webpage has changed since we began this project: (a) 'Recruitment Status' has been renamed 'Status' and (b) Phases 0–4 have been replaced by Early Phase 1 and Phase 1–Phase 4.]

This yielded 112,013 records which were downloaded in two ways:

1. 112,013 XML files (in six lots, based on recruitment type, as indicated in the main Methodology), which are available at https://osf.io/uj7r4/ in six lots; and

2. Six TSV (tab separated values) files which contained a total of 112,013 records (again, in six lots based on recruitment type). Here, each study record was saved as a separate line in the file, with tabs as delimiters between each field. Subsequently each of the six files was saved as an ods file.

Since an XML file has a lot of data for a given clinical trial, we used a python script (named in the box, below) to extract information on the 'Authority'. This field was stitched to the corresponding data in the TSV file to yield a total of 27 columns per record.

| **SCRIPT**  The following script was used at this stage:  S1 Table_ReadAndExtract_Authority.py |
| --- |

**(B) Two cases in the sorting of US- versus non-US authority records**

This note relates to S6–S7 Tables.

To extract trials registered with a US authority, we extracted all trials with the following keywords: “USA:” and “United States:”. However note that

1. NCT00477763 was included even though 'United States' was spelled 'Unitet States', and

2. NCT01976741, that included the word 'usage', was rejected.

**(C) Examples of authorities in the United States where trials were registered.**

This note relates to S6–S7 Tables.

1. United States: Food and Drug Administration
2. United States: Institutional Review Board
3. United States: Federal Government
4. United States: Western Institutional Review Board
5. United States: Data and Safety Monitoring Board
6. USA: Internal Review Board
7. USA: WIRB
8. USA: Walter Reed Army Institute of Research Institutional Review Board
9. USA: National Institutes of Health
10. USA: Food and Drug Administration – Center for Drug Evaluation and Research
11. United States: Women and Infants Institutional Review Board
12. United States: PATH

**(D) Processing the trials for the step of matching 'Last name' and 'Role'**

This note relates to S6–S8 Tables.

The records listed in S6 and S7 Tables have two fields each, ie. NCT ID and Authority. For the next step, ‘Last Name’ and ‘Role’ were extracted from their corresponding XML files using two scripts (named in the box, below) and stitched up to the corresponding rows in these two tables. If an NCT ID had multiple names, then pipes were introduced to separate successive ones. The same thing was done for successive roles. Thus, S8 Table has four columns for each record, ie. NCT ID, Last name, Role and Authority. Data in this table was then sorted to identify nulls in the 'Last name' or 'Role' fields. A record was rejected if it had a 'null' in one or both of these fields.

| **SCRIPTS**  The following two scripts were used at this stage:  1. S8 Table_ReadAndExtract_LastName.py  2. S8 Table_ReadAndExtract_Role.py |
| --- |

**(E) Separating single names and their corresponding roles**

This note relates to S9–S10 Tables.

The data (31,375 records from S9 Table) was sorted by running two scripts (named in the box, below). Records that contained multiple names and roles were segregated such that each row contained one name and the corresponding role. They are listed in the LineByLine sheets in S10 Table.

| **SCRIPTS**  The following two scripts were used at this stage:  S10 Table_NameLineByLine.py  S10 Table_RoleLineByLine.py |
| --- |

**(F) Descriptions of the scripts used to process data**

These scripts relate to S1, S8 and S10 Tables.

1. For each of the NCT IDs listed in S1 Table, the script **S1 Table_ReadAndExtract_authority.py** was run to extract the field ‘Authority’ from the corresponding XML files. This field was then ‘stitched’ to the TSV file to obtain 27 fields of information for each record.
2. The 'NCT ID' column from S6 Table (31,833 records) and S7 Table (3288 records) were merged. Scripts **S8 Table_ReadAndExtract_lastName.py** and **S8 Table_ReadAndExtract_role.py** were run to extract 'Last name' and 'Role' columns and sorted into a 'NameAndRole' sheet in S8 Table for trials that listed both the name of the investigator and corresponding role played by him or her, and a 'Nulls' sheet that listed the remaining records.
3. The data (31,375 records from S9 Table) was sorted into the 'LineByLine' sheet by running scripts **S10 Table_NameLineByLine.py** and **S10 Table_RoleLineByLine.py** where records that contained multiple names and roles were segregated such that each row contains one name and the corresponding role. This data is listed in S10 Table.

**(G) Issues with regard to multiple PIs per trial.**

In the initial phase of this work we identified a very large number of trials with more than one PI per trial. On double checking this data recently, we found that many of those trials now list only one PI per trial. Nevertheless we did find trials that continue to list multiple PIs. The first few examples that we encountered have been listed in the main text.

**(H) Identifying PIs in the history records of a sample of S8 Table rejects and S11 Table rejects.**

In the **S8 Table**, 31,392 trials listed both the investigator's name and role, and, **in the original study,** these were selected to take forward. **3,729 unique NCT IDs** were not selected because they were missing the name or role.

Likewise, in the **S11 table**, there were 60,787 investigators’ ‘names’ that were of a real individual, and, **in the original study,** these were selected to take forward. 10,572 ‘names’ were not selected because they were junk information, and not of a real individual. The 10,572 names can be reduced to **8,907 unique NCT IDs** by removing cases where different ‘names’ belonged to the same NCT ID.

**Totalling the 3,729 and 8,907 rejected records**, from S8 Table and S11 Table respectively, gave us **12,636 unique NCT IDs**. We wished to create a 5% sample, or 632, of these 12,636 records, in proportion to the representation of these IDs in the S8 and S11 Tables. There were approximately twice as many records in the S11 set as in the S8 set, and we therefore took **211** records from the S8 set and **422** of the S11 set.

**[Sampling methodology**: For the S8 and S11 sets, separately, we wished to take a certain number of random samples of NCT IDs. We used the module ‘random’ in python. We used ‘sample method’. It took two arguments (i) size of the population and (ii) size of the data sample to be collected. It returned 633 unique NCT IDs. We sampled without replacement. Total sample size after random sampling from the S8 and S11 sets was 633. The python script used for sampling and scraping was S13 Table_number.py.]

Then we examined **all** the ‘History of Changes’ records on the ClinicalTrials.gov Archive Site for each of these 633 NCT IDs. In every NCT record there was a ‘History of Changes’ link, which went to the relevant ‘ClinicalTrials.gov Archive Site’. This site may have listed many URLs, which provided the details of changes to the record over time. For a given record, the script S13 Table_number.py counted the number of URLs in the history of that trial. The script S13 Table_url.py generated the URL for each step in the history of that trial.

We then used the regex module in the web-scraping function, which looked for exact text that we specified (as mentioned in S13 Table_historyinformation.py). In this case, we specified ‘‘Principal Investigator’ and the text next to it. Therefore, the Principal Investigator information was picked up from anywhere on the page.

The question arose as to which part of the record we should examine, in order to retrieve PI information. There were multiple possibilities:

category (a): Under Sponsor, the company name was listed as PI. (We excluded such cases from our count of trial PIs.)

category (b): Under Sponsor, the Responsible Party was listed and the PI name may have been listed. (We considered this to be an unambiguous PI, and included such cases in our count of trial PIs.)

category (c): Under Contacts/Location, the Study Official may have been listed at the top of this section, and this person may have been the PI. (We considered this to be an unambiguous PI, and included such cases in our count of trial PIs.)

category (d): Under Contacts/Location, there may have been a long list of sites, and each may have listed a PI, without clarity as to which one was the overall PI. In some cases there was a single site, and the PI may have been listed, or two sites with one PI each. (We considered all these cases to be ambiguous about the overall PI for the trial, and excluded such cases from our count of trial PIs.)

Based on these criteria:

Of S8’s 211 NCT IDs, 75 had PI information (S13 Table). Out of the 75 cases,

categories a, b, c and d, had 0, 13, 3, and 59 PIs, respectively. The 59 category d records were 15 + 44, as explained on sheet S8_categorization of 75 NCT IDs.

Thus PI information could be retrieved from the history records of 16/75, which was also 16/211 (7.6%) NCT IDs.

Of S11’s 422 NCT IDs, 54 had PI information (S13 Table). Out of the 54 cases,

categories a, b, c and d, had 4, 0, 9 and 41 PIs, respectively. The 41 category d records were 8 + 33, as explained on sheet S11_Categorization of 21 NCT IDs.

Thus PI information could be retrieved from the history records of 9/54, which was also 9/422 (2%) NCT IDs.

Examining the history of particular NCT IDs did not provide significant information on PIs.

| **SCRIPTS**  The following scripts were used at this stage:  S13 Table_number.py  S13 Table_url.py  S13 Table_historyinformation.py |
| --- |

**(I) Quantifying name ambiguities**

In the manuscript, we listed the categories of variations in names that we identified, as follows (where the categories in bold are discussed further below).

**a) Extraneous information along with the name:**

(i) The name may have had a prefix (examples: Prf.; Prof. Dr.; COL) or **suffix (examples: MD; Jr.; III; M.D., Principal Investigator; BSc, MBCHB, MD, Study Director) of varying lengths**; or

(ii) **there may have been a punctuation mark within the name.**

b) Different kinds of variations of the name:

(i) The name may have had spelling mistakes;

(ii) one or more parts of the name may have been abbreviated or truncated;

(iii) parts of the name may have been ordered differently;

**(iv) the middle name may or may not have been mentioned;**

(v) parst of the name may or may not have been hyphenated;

(vi) the surname may have been modified;

(vii) the surname may have been repeated;

(viii) the person’s initials may or may not have been separated by spaces;

**(ix) the entire name, or parts of it, may have been in capitals;**

(x) the name may have contained a non-English character or the closest English character;

(xi) the first name may have been split into two, or the first and middle name may have been

merged;

(xii) the surname may have been split into two, or the middle and surname may have been merged;

**(xiii) a nickname, in brackets, may have been mentioned in the middle of the name; or**

(xiv) the Americanized nickname of part of a foreign name may have replaced the original.

**c) Other complications with the names:**

(i) **A person’s entire name may have been represented by just one word;** or

(ii) two individuals may have shared the same name.

We used automated methods to quantify or eliminate some of these 18 categories of errors

(as detailed in S14 Table – S18 Table, where S14 Table provides a summary of these five files).

Category a(i). We used a comma as a delimitor to eliminate all suffixes (as described in S15 Table_name_issue_1.py).

Category a(ii). We eliminated all punctuation marks in the middle of the name (as described in S18 Table_name_issue_4.py).

Category b(iv) cannot be readily solved since a middle name (or the first letter of the middle name) may indicate the same person or a different person. This contributed the bulk of the ‘problem cases’ which we used to quantify names which cannot be repurposed.

Category b(ix) was eliminated by converting all caps to lower case before beginning to process the name files.

Category b(xiii). We eliminated any part of the name that was within brackets, as detailed in S18 Table_name_issue_4.py.

Category c(i) Such cases were identified, and were part of the ‘problem cases’ used to calculate how many names could not be repurposed.

Miscellaneous: A mix of categories was estimated based on a sample of records, as detailed in S14 Table.

This work yielded an estimate of 12.8% of names that could not be identified unambiguously. We have not quantified the other categories of errors. However, based on preliminary work, we believe that they are not numerous.

| **SCRIPTS**  The following scripts were used at this stage:  S15 Table_name_issue_1.py  S16 Table_name_issue_2.py  S17 Table_name_issue_3.py  S18 Table_name_issue_4.py |
| --- |

**(J) Whether PI information is available from Responsible Party**

The 1,221 cases with no RP: We used the regex module in the web-scraping function, which looked for the exact text (such as ‘Responsible Party’) which we specified. After screening for ‘Responsible Party’ text on the webpage, 1,221 cases did not have any ‘Responsible Party’ text. The remaining 33,900 NCT records did have the ‘Responsible Party’ text in the NCT record. The web-scraping function S19 Table_main_RP_screen_regex.py was used to screen each NCT ID for ‘Responsible Party’ text.

The RP is usually listed both at the top of the NCT ID record ('top RP’) and at the bottom (‘bottom RP’).

**Top RP:**

Scraping the NCT record for ‘top RP’ was not problematic.

The requests library was used to retrieve content from a webpage of NCT records, and was parsed to an HTML record. Then the beautifulsoup package in python3.0 was used to retrieve RP information from the @id tag of the HTML page. The web-scraping function S19 Table_main_Rptop.py was used for the top RP.

**Bottom RP:**

Scraping the NCT record for ‘bottom RP’ was somewhat problematic.

‘Bottom RP’ information could be present in multiple xpath selectors, such as div 5, div 7, div 8 and div 9.

We used the regex module in the web-scraping function, which looked for the exact text pattern having information of ‘Responsible Party’ at the bottom of the page (the text pattern of the information was: ‘Responsible Party: abcdef’, where abcdef was the name of the responsible party). The S19 Table_main_RP_extract_regex.py programme was used to retrieve this information.

**Summary:**

We preferred to extract RP information from the ‘top RP’. Where such information was missing, we extracted it from ‘bottom RP’.

| **SCRIPTS**  The following scripts were used at this stage:  S19 Table_main_RP_screen_regex.py  S19 Table_main_Rptop.py  S19 Table_main_RP_extract_regex.py |
| --- |
